# Are Antiemetics the Next Therapeutic Strategy against Cisplatin-Associated Acute Kidney Injury?

**DOI:** 10.34067/KID.0000000000000518

**Published:** 2024-08-29

**Authors:** Shveta S. Motwani, Ala Abudayyeh

**Affiliations:** 1 Dana-Farber Cancer Institute, Harvard Medical School, Boston, Massachusetts; 2 MD Anderson Cancer Center, University of Texas, Houston, Texas

**Keywords:** AKI, cisplatin, cisplatin nephrotoxicity

Cisplatin remains a cornerstone in the arsenal against solid organ malignancies, offering hope to many battling cancers. However, its benefits come with significant risks, notably nephrotoxicity, which affects up to 30% of patients receiving this chemotherapy. The etiology of AKI is often clinically determined and is commonly attributed to hypovolemia from gastrointestinal toxicity and to direct tubular toxicity from cisplatin. The near-universal protocolized administration of intravenous fluids (IVFs) and antiemetics before cisplatin has helped mitigate these toxicities significantly.

The current study, the largest of its kind, retrospectively evaluated 6889 patients from the Memorial Sloan Kettering Cancer Center, offering data on the incidence of AKI in the context of cisplatin and serotonin antagonist (5-HT_3_RA) coadministration.^1^ Furthermore, they sought to assess any differential effects of first- and second-generation 5-HT_3_RA. This was based on the concern raised about 5-HT_3_RA potentiating the nephrotoxic effect of cisplatin by inhibiting multidrug and toxin extrusion protein.^2^ Multidrug and toxin extrusion protein is a proximal tubular epithelial brush border renal transporter responsible for cisplatin excretion from the cell, and its inhibition leads to increased intracellular cisplatin accumulation and toxicity. Second-generation 5-HT_3_RA reportedly had less adversarial effects on toxic accumulation of cisplatin compared with their first-generation counterparts.

The effect of 5-HT_3_RA on kidney function remains elusive. While the abovementioned preclinical studies have raised concerns about increased risk of cisplatin-associated AKI, other studies have suggested the contrary. For example, in the critical care literature, use of ondansetron is associated with a lower all-cause mortality among critically ill patients with AKI.^3–5^ In one of these studies,^4^ on the basis of comparison of gene expression signatures, the authors hypothesized that the therapeutic effect of ondansetron may be elicited through the NF-kappaB and Janus kinases-signal transducers and activators of transcription proteins pathways, which are implicated in the pathophysiology of AKI. Thus, this protective effect underscores the critical role of antiemetics not just in improving patient comfort but also in possibly safeguarding renal function.

It is important to note that, in the present study, all patients received 5-HT_3_RA before cisplatin and only those who were symptomatic received it after cisplatin. Thus, both groups had some exposure to 5-HT_3_RAs. In addition, given the retrospective nature of the study, it is not clear whether the patients in the non–5-HT_3_RA group continued to take these, as needed, at home during the 30-day post-cisplatin window for AKI. Nevertheless, the findings are compelling: Patients receiving any 5-HT_3_RA therapy exhibited a 16% reduction in the odds of developing AKI. However, this could also be because of indication bias as patients with moderate to severe gastrointestinal toxicity will often receive additional IVFs as part of supportive care. This additional IVF could also be responsible for reduced risk of AKI in the 5-HT_3_RA group. The data regarding IVF administration and whether these were different between groups were not included in the study.

In conclusion, despite the strengths of this study, including its large size, robust analysis, and strong clinical rationale, there are critical limitations that need to be addressed. The retrospective design inherently carries biases, including selection bias and indication bias, which can affect the validity of the findings. In addition, the study's reliance on electronic health records may have led to misclassification or incomplete capture of data, particularly for patients treated with multiple antiemetics or those receiving care outside of the electronic system. These factors could have influenced the observed protective effect of 5-HT_3_RA, necessitating cautious interpretation of the results. The findings overall reassuring regarding the adverse effects of cisplatin and 5-HT_3_RA coadministration. The beneficial effects of 5-HT_3_RA, however, need to be confirmed in randomized clinical trials. This study has certainly helped moved the needle forward and taken this important step of examining the data in patients with cancer where these antiemetics are used liberally in daily practice.

Future studies should prospectively compare cisplatin-treated groups and have meticulous account of the amount of IVF administered, allowing comparison between groups. It would also be helpful to see a dose-response relationship between 5-HT_3_RA and changes in creatinine. Randomized controlled trials in the cancer population will also help determine whether 5-HT_3_RA can be used as a therapeutic strategy for the purpose of prevention and treatment of AKI in the absence of symptom-based indications.
